# Predictive value of inflammatory factors on coronary restenosis after percutaneous coronary intervention in patients with coronary heart disease: A protocol for systematic review and meta-analysis

**DOI:** 10.1097/MD.0000000000025356

**Published:** 2021-04-02

**Authors:** Xin Chu, Ruzhu Wang, Guixian Song, Xiaohan Jiang

**Affiliations:** a Department of Cardiology; b Department of Medical Genetics and Prenatal Diagnosis, Hospital Affiliated 5 to Nantong University, Taizhou People's Hospital, Taizhou 225300, Jiangsu province, China.

**Keywords:** biomarker, coronary heart disease, coronary restenosis, diagnosis, inflammatory factors, meta-analysis, percutaneous coronary intervention

## Abstract

**Background:**

Evidence reveals that inflammatory factors can predict coronary restenosis in patients suffering from coronary heart disease (CHD) after percutaneous coronary intervention (PCI). Perhaps, inflammatory factors are promising biomarkers for the diagnosis of coronary restenosis after PCI. However, the accuracy of inflammatory factors has not been systematically evaluated. Therefore, it is necessary to perform a meta-analysis to certify the diagnostic values of inflammatory factors on coronary restenosis after PCI.

**Methods:**

China National Knowledge Infrastructure (CNKI), Wanfang, VIP, China Biology Medicine disc (CBM), PubMed, EMBASE, Cochrane Library and Web of Science were searched for relevant studies to explore the potential diagnostic values of inflammatory factors on coronary restenosis after PCI from inception to January 2021. All data were extracted by 2 experienced researchers independently. The risk of bias about the meta-analysis was confirmed by the Quality Assessment of Diagnostic Accuracy Studies-2 (QUADAS-2). The data extracted were synthesized and heterogeneity was investigated as well. All of the above statistical analyses were carried out with Stata 16.0.

**Results:**

The results of this meta-analysis will be submitted to a peer-reviewed journal for publication.

**Conclusion:**

This study clarified confusions about the specificity and sensitivity of inflammatory factors on coronary restenosis after PCI, thus further guiding their promotion and application.

**Ethics and dissemination:**

Ethical approval will not be necessary since this systematic review and meta-analysis will not contain any private information of participants or violate their human rights.

**Trial Registration Number:**

DOI 10.17605/OSF.IO/N28JX.

## 1 Introduction

In recent years, with the advent of an aging society and the improvement of living standards, the incidence and mortality of coronary heart disease (CHD) have gradually increased, which poses a serious threat to the health of people.^[1–3]^ Percutaneous coronary intervention (PCI) is the most commonly adopted method for the treatment of CHD.^[4,5]^ At present, it has been recommended by a number of cardiovascular diagnoses and treatment guidelines at home and abroad.^[6–8]^ Because of its minimally invasive treatment, it is easier to be accepted by patients than coronary artery bypass grafting.

However, coronary restenosis is easy to occur after PCI, the incidence rate can reach 5% to 20%, and cardiovascular events occur, which affects the therapeutic effects.^[9–11]^ Reexamination through coronary angiography (CAG) is the criterion standard for the diagnosis of coronary restenosis after PCI.^[12,13]^ However, CAG is a diagnostic method with high cost, complex operation, and serious complications. As a screening method for coronary restenosis after PCI, it is greatly limited clinically. Therefore, there is an urgent need for a minimally invasive and inexpensive method for primary screening, and further CAG examination is recommended for patients with potential coronary restenosis.

At present, it is well known that a variety of inflammatory factors are involved in the whole process of coronary heart disease, and the increase of inflammatory markers is significantly related to the increase of mortality in patients with CHD.^[14]^ Inflammatory factors are considered as important factors in the occurrence and development of CHD. The vascular stent implanted after PCI produces lumen hyperplasia on the basis of a series of pathophysiological changes, and finally forms coronary restenosis.^[15]^ The most important pathological process is inflammatory reaction, which has been widely accepted by academic circles.^[16]^ These inflammatory markers include neutrophil/lymphocyte ratio (NLR), interleukin-6 (IL-6), hypersensitive C-reactive protein (Hs-CRP), plasma lipoprotein-associated phospholipase A2 (Lp-PLA2), homocysteine (Hcy), and so on.^[17–24]^ At the same time, these inflammatory markers are cheap and easy to be obtained. Therefore, it is expected to become a potential indicator for preliminary laboratory screening and diagnosis of coronary restenosis.

However, the relationship between inflammatory factors and coronary restenosis after PCI is still controversial.^[17–24]^ To clarify the value of inflammatory factors in terms of predicting coronary artery restenosis after PCI in patients with CHD, we conducted a meta-analysis.

## 2 Methods

### 2.1 Study registration

The protocol of the systematic review has been registered on Open Science Framework (registration number: DOI 10.17605/OSF.IO/N28JX). It was reported by the following guideline of Preferred Reporting Items for Systematic Reviews and Meta-analysis Protocol statement.^[25]^

### 2.2 Inclusion criteria for study selection

#### 2.2.1 Type of studies

To explore the diagnostic value of inflammatory factors on coronary restenosis after PCI.

#### 2.2.2 Type of participants

All patients with CHD after PCI were included.

#### 2.2.3 Type of index test

Index test: inflammatory factors were applied to detect patients with coronary restenosis after PCI. However, we excluded case reports, reviews, cell, or animal studies.

#### 2.2.4 Outcome measurements

Outcomes include pooled sensitivity (SEN), specificity (SPE), positive likelihood ratio (PLR), negative likelihood ratio (NLR), diagnostic odds ratio (DOR), area under the curve (AUC), and their 95% confidence intervals (CIs).

### 2.3 Data sources and search strategy

This study conducted a literature search in China National Knowledge Infrastructure (CNKI), Wanfang, VIP, China Biology Medicine disc (CBM), PubMed, EMBASE, Cochrane Library, and Web of Science. We made a final search in January 2021. The search strategy of Pubmed is displayed in Table 1.

**Table 1:** PubMed search strategy.

Number	Search terms
#1	Coronary Disease[MeSH]
#2	Coronary Heart Disease[Title/Abstract]
#3	Coronary Diseases[Title/Abstract]
#4	Coronary Heart Diseases[Title/Abstract]
#5	Disease, Coronary[Title/Abstract]
#6	Disease, Coronary Heart[Title/Abstract]
#7	Diseases, Coronary[Title/Abstract]
#8	Diseases, Coronary Heart[Title/Abstract]
#9	Heart Disease, Coronary[Title/Abstract]
#10	Heart Diseases, Coronary[Title/Abstract]
#11	or/1–10
#12	Percutaneous Coronary Intervention[MeSH]
#13	Percutaneous Coronary Revascularization[Title/Abstract]
#14	Coronary Intervention, Percutaneous[Title/Abstract]
#15	Coronary Interventions, Percutaneous[Title/Abstract]
#16	Coronary Revascularization, Percutaneous[Title/Abstract]
#17	Coronary Revascularizations, Percutaneous[Title/Abstract]
#18	Intervention, Percutaneous Coronary[Title/Abstract]
#19	Interventions, Percutaneous Coronary[Title/Abstract]
#20	Percutaneous Coronary Interventions[Title/Abstract]
#21	Percutaneous Coronary Revascularizations[Title/Abstract]
#22	Revascularization, Percutaneous Coronary[Title/Abstract]
#23	Revascularizations, Percutaneous Coronary[Title/Abstract]
#24	or/12–23
#25	Coronary Restenosis[MeSH]
#26	Coronary Restenoses[Title/Abstract]
#27	Restenoses, Coronary[Title/Abstract]
#28	Restenosis, Coronary[Title/Abstract]
#29	or/25–28
#30	Marker[Title/Abstract]
#31	Biomarker[Title/Abstract]
#32	or/30–31
#33	diagnos^∗^[Title/Abstract]
#34	sensitivity[Title/Abstract]
#35	specificity[Title/Abstract]
#36	ROC curve[Title/Abstract]
#37	or/33–36
#38	#11 and #24 and #29 and #32 and #37

### 2.4 Data collection and analysis

#### 2.4.1 Study selection

**Figure 1:**
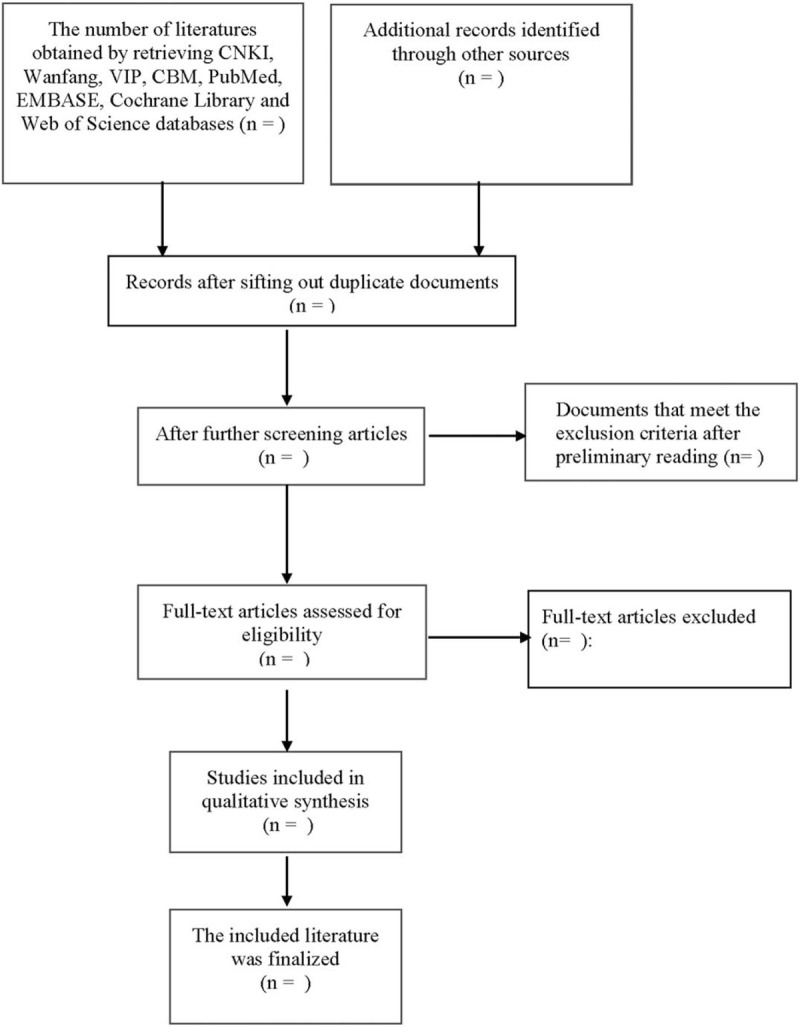
Flow diagram of literature retrieval.

Two researchers independently complete the literature screening, exclude the studies that obviously do not meet the inclusion criteria, and further read the abstracts and the full texts to determine whether they meet the inclusion criteria. The data included in the literature will be extracted and cross-checked. Disagreement should be solved by consulting a third researcher, so as to reach a consensus. The screening flow chart of this study is demonstrated in Figure 1.

#### 2.4.2 Data extraction

The data extraction form includes the following items: first author, publication year, regions, sample size, sample types, control group, inflammatory factors detection methods, and data needed for diagnostic meta-analysis.

#### 2.4.3 Dealing with missing data

If the data of the required study are incomplete or not reported in the study, the researcher will contact the first author or other authors of the study by phone or email. If the required data are not available, we will perform descriptive analysis, instead of meta-analysis, and exclude these studies if necessary.

### 2.5 Quality assessment

The methodological quality of the included studies was assessed by the Quality Assessment of Diagnostic Accuracy Studies-2 (QUADAS-2) criteria. Two reviewers independently and blindly checked the studies. Any discrepancies between the 2 reviewers were resolved by consensus after discussion.

### 2.6 Statistical analysis

All of the above statistical analyses were performed with Stata 16.0 (StataCorp LLC, college station, TX). We calculated the pooled SEN, SPE, PLR, NLR, DOR, and their 95% CI. In addition, the pooled diagnostic value of inflammatory factors through the summary receiver operating characteristic curve (SROC) and AUC was tested. The threshold effects were detected by using spearman correlation coefficient. The calculation of heterogeneity was caused by the nonthreshold effects of Cochrane-Q and *I*^2^ values, and a fixed-effect model (without obvious inhomogeneity) or a random effects model (with significant heterogeneity) was employed to merge the data. The statistical test level was α = 0.05.

### 2.7 Subgroup analysis

To further investigate potential heterogeneity, subgroup analyses were conducted based on ethnicity, the source of inflammatory factors, and sample size.

### 2.8 Sensitivity analysis

To test the stability of the meta-analysis results, we will adopt the one-by-one exclusion method to analyze the sensitivity of the results.

### 2.9 Reporting bias

The Deeks symmetry test was performed to detect whether there is a publication bias in the included studies.

### 2.10 Ethics and dissemination

Since the program does not include the recruitment of patients and the collection of personal information, it does not require the approval of the Ethics Committee.

## 3 Discussion

CAG examination is the “criterion standard” for the judgment of coronary restenosis after PCI, but CAG is an invasive examination and the compliance of patients is low.^[26,27]^ Cardiovascular clinical attention is focused on finding serum markers that can effectively and easily reflect coronary restenosis after PCI. Therefore, some researchers have suggested that the detection of serum inflammatory markers may be more effective in predicting coronary restenosis after PCI.

Coronary restenosis is characterized by a gradual reduction of coronary artery lesions with stents due to arterial injury and neointimal hyperplasia.^[28,29]^ On the basis of a previous study, restenosis occurs in about 30% of CHD patients underwent PCI with bare-metal stents.^[29,30]^ The occurrence and development of restenosis is closely related to the inflammatory activity of endothelial cells.^[23,31]^ Inflammatory factors can promote the formation of coronary artery disease and the proliferation of neointima in patients with CHD through a variety of functions, and promote the proliferation / migration of smooth muscle cells to intima.^[32,33]^ Sun et al revealed that IL-6 is an independent risk factor for restenosis, and AUC is >0.6.^[17]^ Most importantly, when IL-6 is combined with other inflammatory factors, the AUC is >0.9.^[17]^

Through this meta-analysis, we will screen out the inflammatory factors with high sensitivity and specificity or the best combined inflammatory markers. This will provide a new theoretical basis for the detection, prevention, and treatment of coronary restenosis after PCI.

## Author contributions

**Conceptualization:** Xin Chu.

**Data collection:** Ruzhu Wang and Guixian Song.

**Data curation:** Xin Chu, Ruzhu Wang.

**Formal analysis:** Guixian Song.

**Funding acquisition:** Xin Chu.

**Funding support:** Xin Chu.

**Project administration:** Xin Chu.

**Resources:** Guixian Song, Xin Chu.

**Software operating:** Xiaohan Jiang and Ruzhu Wang.

**Software:** Guixian Song, Xiaohan Jiang.

**Supervision:** Guixian Song, Xiaohan Jiang.

**Validation:** Xiaohan Jiang.

**Visualization:** Xiaohan Jiang.

**Writing – original draft:** Xin Chu and Ruzhu Wang.

**Writing – review & editing:** Xin Chu and Ruzhu Wang.
